# Current state of cancer immunity cycle: new strategies and challenges of using precision hydrogels to treat breast cancer

**DOI:** 10.3389/fimmu.2025.1535464

**Published:** 2025-03-07

**Authors:** Yingze Zhu, Yanlin Su, Yaxin Guo, Xinyue Wang, Zhuoqi Zhang, Yige Lu, Hang Yang, Hui Pang

**Affiliations:** ^1^ Department of Oncology, Harbin Medical University Cancer Hospital, Harbin, China; ^2^ Department of Gastroenterology and Hepatology, Tianjin Second People’s Hospital, Tianjin, China

**Keywords:** hydrogel, breast, cancer, immune, therapy

## Abstract

The cancer-immunity cycle provides a framework for a series of events in anti-cancer immune responses, initiated by T cell-mediated tumor cell killing, which leads to antigen presentation and T cell stimulation. Current immunomodulatory therapies for breast cancer are often associated with short duration, poor targeting to sites of action, and severe side effects. Hydrogels, with their extracellular matrix-mimicking properties, tunable characteristics, and diverse bioactivities, have garnered significant attention for their ability to locally deliver immunomodulators and cells, providing an immunomodulatory microenvironment to recruit, activate, and expand host immune cells. This review focuses on the design considerations of hydrogel platforms, including polymer backbone, crosslinking mechanisms, physicochemical properties, and immunomodulatory components. The immunomodulatory effects and therapeutic outcomes of various hydrogel systems in breast cancer treatment and tissue regeneration are highlighted, encompassing hydrogel depots for immunomodulator delivery, hydrogel scaffolds for cell delivery, and immunomodulatory hydrogels dependent on inherent material properties. Finally, the challenges that persist in current systems and future directions for immunomodulatory hydrogels are discussed.

## Graphical Abstract

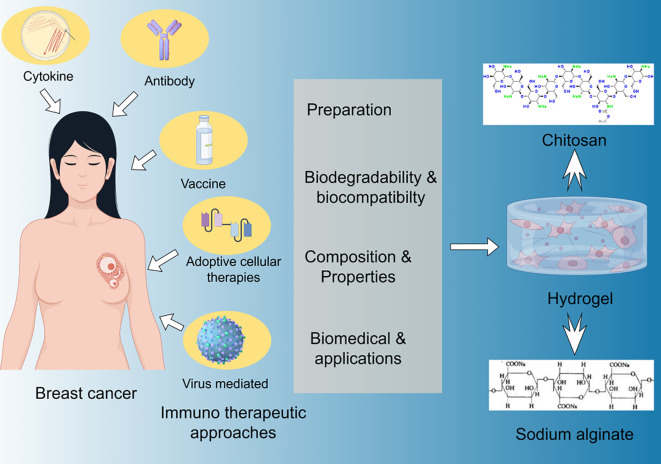

## 1 Introduction

Breast cancer, one of the most prevalent malignancies among women globally, has emerged as a significant challenge in contemporary medicine. It is a cancer that initially develops from the malignant proliferation of lobular or ductal cells (1). According to the latest data from the World Health Organization, approximately 2.3 million new cases of breast cancer were diagnosed worldwide in 2020, accounting for 11.7% of all new cancer cases (2). Despite advancements in diagnostic and treatment technologies in recent years, breast cancer remains one of the leading causes of cancer-related deaths among women, resulting in approximately 685,000 female deaths globally in 2020 (3). The molecular spectrum and phenotypic characteristics of breast cancer are diverse. Clinical classification is based on hormone receptor expression, including estrogen receptor (ER), progesterone receptor (PR), and human epidermal growth factor receptor 2 (HER2). This classification system categorizes breast cancer into HER2+, luminal A (HER2-, ER+ and/or PR+), luminal B (HER2+, ER+ and/or PR+), and triple-negative breast cancer (basal-like) subtypes (4–6). Different breast cancer subtypes exhibit distinct clinical features and treatment responses, underscoring the complexity in accurate diagnosis and effective treatment of breast cancer patients (7). Moreover, traditional treatment modalities such as chemotherapy and radiotherapy lack precision, often leading to adverse effects including edema in normal tissues, gastrointestinal symptoms, fatigue, and hair loss (8–10). These concerns highlight the urgent need for developing more effective therapeutic strategies.

Traditional breast cancer treatments, such as surgery, radiotherapy, and chemotherapy, while effective in certain cases, are often associated with severe side effects and risk of recurrence (9, 11, 12). In recent years, with the deepening understanding of tumor immunology, immunotherapy has emerged as a new frontier in cancer treatment. However, the efficacy of immunotherapy in breast cancer remains limited, primarily due to the immunosuppressive microenvironment and immune evasion mechanisms of breast cancer (5, 13).

The concept of the cancer-immunity cycle, proposed in recent years, has provided a theoretical framework for understanding tumor immune responses and developing novel immunotherapeutic strategies (14). This cycle encompasses several key steps: following the release of neoantigens by cancer cells, antigen-presenting cells capture and process antigens, subsequently activating T cells. The activated T cells then migrate to and infiltrate the tumor site, killing cancer cells and releasing more tumor antigens, thus restarting the cycle. Within this process, T cells participate in a series of iterative events, forming an immune microenvironment vortex within the cancer-immunity cycle. Studies have shown that the progression of T cell exhaustion spans from progenitor exhausted T cells with stem cell-like properties to terminally exhausted T cells that have lost replicative and effector functions (15, 16). During this process, they produce CXCL13 and form tertiary lymphoid structures, which, due to their lack of capsules, become crucial for anti-tumor immunity (17–19). Simultaneously, the immunosuppressive functions within the tumor immune microenvironment (TIME) drive the exhaustion of tumor-specific CD8+ T cells. Terminally exhausted T cells (Texterm) can produce effector molecules at the transcriptional level, leading to their numerical advantage in the immune microenvironment. Liu et al. demonstrated that CXCL13^+^ CD8^+^ Tex could effectively predict the efficacy of immunotherapy (20).

In the face of these challenges, precision hydrogel technology has garnered widespread attention from researchers in recent years. Hydrogels are three-dimensional network polymers formed by the crosslinking of highly hydrophilic polymer chains in water. Due to the presence of crosslinking systems, they can swell and retain large amounts of water molecules, with water content potentially reaching up to 99% (21). As the degree of crosslinking increases, water absorption capacity and deformability decrease (22, 23). During the dehydration process, both softening and hardening of the hydrogel can occur simultaneously (24). As a highly tunable biomaterial, precision hydrogels have the potential to mimic the extracellular matrix, deliver drugs in a targeted manner, and modulate the immune microenvironment (25). These characteristics make them ideal carriers for enhancing the cancer-immunity cycle and improving the efficacy of immunotherapy. In recent years, researchers have designed smart hydrogels responsive to the tumor microenvironment, enabling precise release of immunomodulators. The reactive oxygen species (ROS) generated can polarize tumor-associated macrophages (TAMs) to M1-TAMs, induce T cell infiltration and activation, while upregulating PD-L1 expression (26, 27). Furthermore, numerous liposome-hydrogel composites have been tested in various cell and animal models, with future studies needed to further investigate their clinical applications for patient benefit (28).

This review aims to comprehensively explore the latest advancements in the cancer-immunity cycle, with a particular focus on the innovative applications of precision hydrogels in enhancing immunotherapy for breast cancer. We will conduct a detailed analysis of current challenges and discuss future research directions, with the goal of providing new perspectives and insights for the development of more effective immunotherapeutic strategies for breast cancer.

## 2 History and milestones in the use of hydrogels for breast cancer immunotherapy

The application of hydrogels in breast cancer immunotherapy represents a significant advancement in targeted drug delivery and personalized medicine.

The evolution of this field can be traced back to the pioneering work of Wichterle and Lim in the 1960s, who first synthesized hydrophilic gels for biological use (29). It wasn’t until the early 2000s that hydrogels began to be explored specifically for cancer immunotherapy (30, 31). A seminal study by Hori et al. demonstrated the potential of injectable hydrogels to deliver immunomodulatory factors in a controlled manner, marking a crucial milestone (32). Subsequently, the work of Mooney and colleagues showcased the ability of alginate hydrogels to act as synthetic immune priming centers, revolutionizing our approach to cancer vaccines (33). In the context of cancer, a landmark study by Yu et al. utilized a novel hydrogel-based platform to deliver checkpoint inhibitors, significantly enhancing the efficacy of immunotherapy in preclinical models (34). In recent years, researchers have developed smart hydrogel systems capable of responding to the tumor microenvironment, representing a paradigm shift in adaptive immunotherapy strategies (35–38). Furthermore, researchers reported a DNA hydrogel for non-destructive isolation of EXOs from complex biological media, enabling direct use of isolated EXOs for precise detection of human breast cancer in clinical samples (39). Gong et al. discovered that umbilical cord blood natural killer (UCB-NK) cell immunotherapy is a promising strategy for cancer treatment. Subsequently, they designed an injectable hydrogel for the co-delivery of suberoylanilide hydroxamic acid (SAHA) and 3-methyladenine (3MA) to enhance the efficacy of UCB-NK cell infusion in TNBC (40). This hydrogel can be injected into the tumor resection bed and form a stable gel *in situ*, thereby achieving pH-sensitive sustained release of SAHA and 3MA. This successfully overcame the bottleneck of easy recurrence and poor prognosis after breast cancer surgery. These milestones underscore the rapid progression and immense potential of hydrogel-based approaches in revolutionizing cancer immunotherapy, paving the way for more effective and personalized treatment modalities.

## 3 Recent advances in the cancer-immunity cycle

### 3.1 Antigen release and presentation

Our understanding of antigen release and presentation has undergone revolutionary changes, transforming the cancer-immunity cycle paradigm. The process of immunogenic cell death (ICD) has emerged as a critical initiator of anti-tumor immunity.

Key characteristics of ICD include the release of damage-associated molecular patterns (DAMPs) such as calreticulin, ATP, and HMGB1 (41). These DAMPs not only attract and activate dendritic cells (DCs) but also enhance their antigen processing capabilities (42). Notably, the discovery of tumor-derived DNA activating the cGAS-STING pathway has revealed new mechanisms of innate immune sensing within the tumor microenvironment (43). Furthermore, recent studies have elucidated the role of extracellular vesicles, particularly exosomes, in shuttling tumor antigens to DCs, thus expanding the scope of antigen presentation beyond traditional cellular mechanisms (44). Moreover, the elucidation of cross-presentation pathways in DCs, especially the role of WDFY4 protein in facilitating antigen transport to the cytosol, has provided new targets for enhancing anti-tumor immunity (45). In the cancer-immunity cycle, the two core steps of cancer antigen presentation to T cells and T cell activation primarily occur within lymph nodes (LNs). In tumor tissues, antigen-presenting cells often display immature and immunosuppressive states, leading to insufficient activation and impaired recruitment of effector T cells. Pyroptosis, as a form of programmed cell death, can induce potent immunogenic cell death, thereby enhancing dendritic cell maturation and forming a positive feedback loop in the cancer-immunity cycle, which maximizes the response rate to immune checkpoint blockade (14). These developments collectively emphasize the complex interactions between dying tumor cells, the innate immune system, and adaptive immunity, offering promising strategies for overcoming immune evasion and improving the efficacy of cancer immunotherapies.

### 3.2 T cell priming and activation

Recent advances in our understanding of T cell priming and activation have significantly refined the cancer-immunity cycle paradigm. The traditional model of T cell activation via the three-signal hypothesis has been expanded to incorporate metabolic reprogramming as a crucial fourth signal.

Notably, the discovery of CD28-independent metabolic stimulation pathways (such as the ICOS-ICOSL axis) has broadened our understanding of T cell activation mechanisms (46). Furthermore, elucidation of the role of the actin cytoskeleton in T cell receptor (TCR) microcluster formation and its subsequent impact on signal transduction has provided new insights into the spatial organization of T cell activation (47, 48). Recent studies have also highlighted the importance of innate-like T cells, particularly mucosal-associated invariant T (MAIT) cells and γδ T cells, in bridging innate and adaptive immunity within the tumor microenvironment (49). Moreover, T cell exhaustion transitions from TEXprog cells with reduced cytokine production to TEXterm cells with increased CXCL13 (50). A subset of Texprog expresses T cell factor 1 (TCF1), exhibiting weak cytotoxicity but strong self-renewal and differentiation capabilities. It is considered the host of Texterm, capable of replenishing tumor-specific T cells, thereby maintaining immune defense composed of tumor-specific T cells. This cell population is recognized as a key barrier to immunotherapy, with strategies aimed at restoring Tex function through immune checkpoint blockade (ICB) and other immunotherapeutic approaches. Notably, ICB can effectively rely on the chemokine receptor CXCR5 expressed by Texprog to promote tumor infiltration and response to checkpoint blockade (51) (**Table 1**).

**Table 1:** T cells in immune microenvironment and breast cancer.

Immune cell	Annotation	Gene signature	Expression of inhibitory receptor	Differentiation trajectory	Functional characteristics	^Ref^
CD8+ T cell	Exhausted T cells	PDCD1	High	Cytotoxic T cells	Chemoattractive, and cytokine signaling	(52)
		GZMB	High	Cytotoxic T cells	Cytokine signaling	(53)
		TNFRSF9	High	Cytotoxic T cells	Systematic enrichment of immunomodulatory	(54)
		CTLA4	High	Cytotoxic T cells	Control T cell activation and tolerance	(55)
		LAG3	High	Cytotoxic T cells	Drive T cell exhaustion, obstructing autocrine IFN-γ dependent anti-tumor immunity	(56)
		CXCL13	High	Cytotoxic T cells	Dysfunctional state, markers of T cell exhaustion	(57)
		HAVCR2	High	Cytotoxic T cells	Immune escape	(52)
		VCAM1	High	Cytotoxic T cells	Transendothelial migration	(58)
CD4+ T cells	Tfh cells	NMB	High	NA	Correlated with plasma cell but not B cell	(59)
		CXCL13	High	NA	Markers of T cell exhaustion	(57)
		PDCD1	High	NA	Regulating T cell function	(60)
		NR3C1	High	NA	Encoding glucocorticoid receptors	(61)

**Figure 1:**
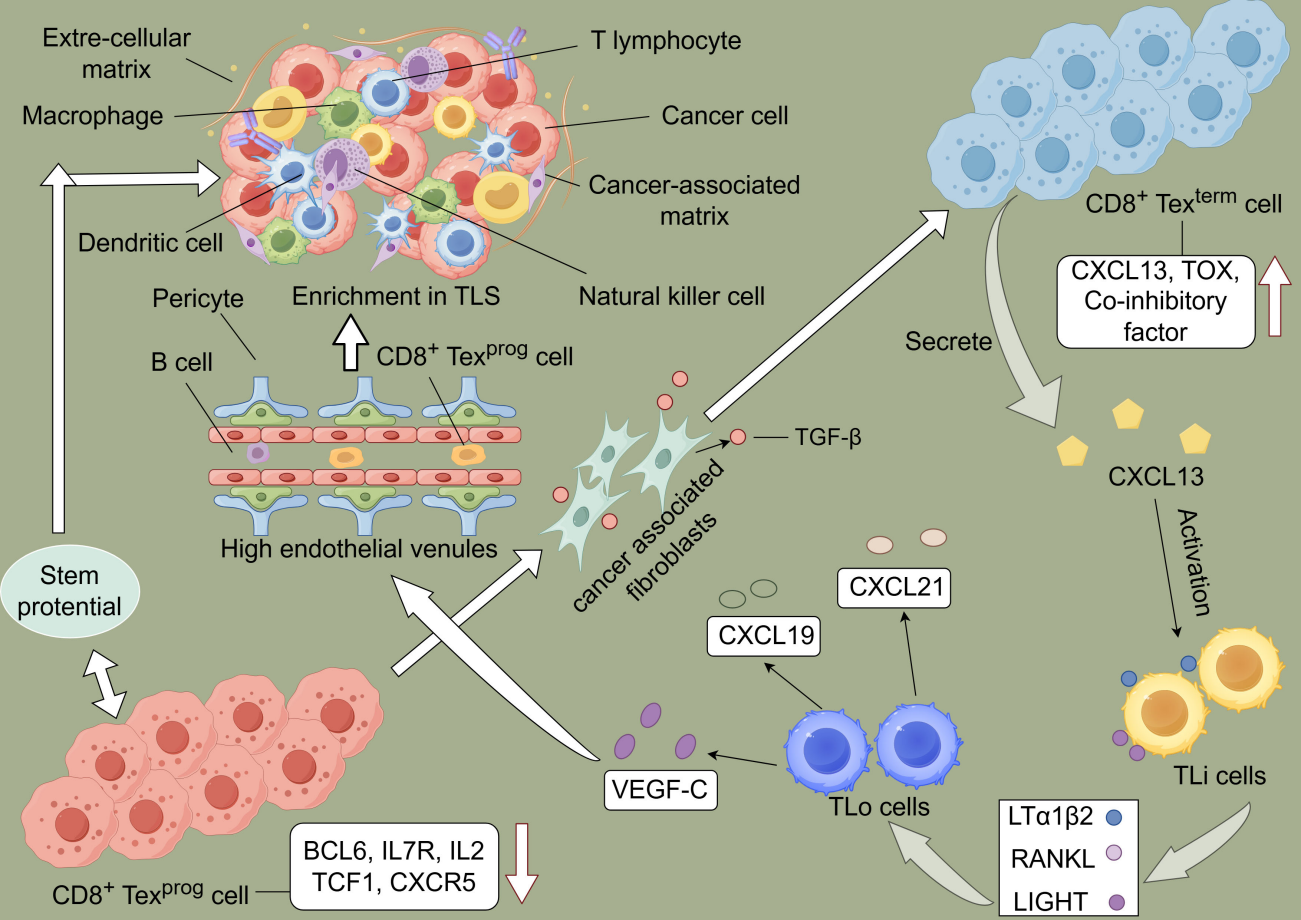
Schematic representation of the immune cycle and T cell exhaustion driving the formation of tertiary lymphoid structures (TLS) within tumors. During the exhaustion process, T cells upregulate and downregulate the expression of related molecules. Subsequently, T cells progress from a Texprog to a Texterm state, characterized by high expression of CXCL13. Lymphoid tissue inducer (LTi) cells express molecules such as LIGHT, which induce lymphoid tissue organizer (LTo) cells to release chemokines, thereby promoting lymphocyte aggregation and the formation of high endothelial venules (HEVs). This process facilitates the formation of TLS within tumors, enabling tumor control.

The discovery of T cell plasticity and the role of epigenetic modifiers in regulating T cell fate decisions have opened new avenues for therapeutic interventions (**Figure 1**) (62). Collectively, these findings underscore the complexity and dynamism of T cell priming and activation in the cancer context, offering promising targets for enhancing the efficacy of cancer immunotherapies and overcoming immune evasion mechanisms.

### 3.3 T cell trafficking to tumors

T cell trafficking to tumor sites is closely associated with chemokine expression and the immune microenvironment. Recent research advances have revealed the complex mechanisms underlying this process, highlighting its intricacy and potential for therapeutic intervention.

The traditional model of T cell recruitment mediated by tumor cell-secreted chemokines such as MIG (CXCL9) and IP-10 has been expanded to include the role of metabolic reprogramming in regulating T cell migration (63, 64). Huang et al. discovered that Th1 and CTL cells in the peripheral blood of breast cancer patients highly express RGS1, whose transcription is regulated by the IFNγ-STAT1 pathway. Activation of this pathway can transcribe immune checkpoint molecules such as PD-L1 and IDO, as well as induce the expression of numerous chemokines, thereby inhibiting T cell migration (65). Notably, Chowdhury et al. demonstrated that PGC1α-mediated mitochondrial biogenesis enhances T cell migration to tumors by increasing fatty acid oxidation (66). Tumor vasculature, long considered a barrier to T cell infiltration, has now been further elucidated as a dynamic regulator of T cell entry. Rigamonti et al. found that normalizing tumor vasculature by inhibiting the Ang2-Tie2 axis can significantly improve T cell infiltration and response to immunotherapy (67). Byrne et al. reported that CD8+ TRM-like cells express high levels of immune checkpoint molecules, including PDCD1 (PD-1), CTLA4, HAVCR2 (TIM-3), and LAG3 (68). Virassamy et al. discovered that the gene signatures of CD8+ TRM cells in triple-negative breast cancer patients are significantly associated with their response to pembrolizumab, highlighting the critical role of these cells in maintaining long-term anti-tumor immunity and their potential as targets for breast cancer vaccines (69). The role of the tumor microenvironment in regulating T cell migration has been further refined, with Lee et al. demonstrating that TGF-β signaling in fibroblasts produces a “T cell exclusion” phenotype, suggesting new targets for combination therapies (70).

These findings collectively emphasize the multifaceted nature of T cell trafficking in cancer and offer promising avenues for enhancing the efficacy of immunotherapies through targeted manipulation of migration pathways.

### 3.4 T cell infiltration into the tumor microenvironment

T cell infiltration into the tumor microenvironment (TME) has emerged as a critical determinant of immunotherapy efficacy and patient prognosis. Recent research advances have unveiled the complex interactions between T cells and the TME, revealing novel mechanisms and potential therapeutic targets.

Notably, Jerby-Arnon et al., utilizing single-cell RNA sequencing, elucidated T cell exclusion programs in tumors, identifying that CDK4/6 inhibition can enhance T cell infiltration, reverse resistant cell states, and induce SASP components (71). The metabolic status of the TME has been recognized as a major obstacle to T cell function, with hypoxia in the TME inducing immunosuppression and inhibiting the cytotoxic function of NK cells. Murphy et al. demonstrated that targeting glutamine and aspartate metabolism can reshape the TME and improve immune cell infiltration (72). Furthermore, Honda et al. highlighted the role of the extracellular matrix (ECM) in T cell exclusion, revealing that capsular tissue protein induced in CD8+ T cell exclusion zones within TGF-β signaling-rich areas forms a fibrotic barrier, impeding T cell entry. Additionally, EMILIN1, a TGF-β inhibitor upregulated in IFNγ-iCAFs, directly modulates the immunosuppressive function of TGF-β (73). This suggests ECM modulation as a potential adjunctive strategy. Gandhi et al. emphasized the importance of innate immune cells in facilitating T cell infiltration, revealing that dendritic cells producing CXCL9/CXCL10 are crucial for effective T cell recruitment. Moreover, Zhang et al. uncovered a novel mechanism whereby hypoxia-induced ANGPTL4 promotes an immunosuppressive TME by inhibiting T cell infiltration and activation. Concurrently, researchers found that high levels of CAF infiltration may attenuate the efficacy of immunotherapy, providing a new target for combination therapies (74).

These findings collectively underscore the multifaceted nature of T cell infiltration in the TME and highlight promising avenues for enhancing the efficacy of immunotherapies through targeted manipulation of the tumor ecosystem.

### 3.5 T cell recognition of cancer cells

T cell recognition of cancer cells is a critical step in the cancer-immunity cycle, and recent research advances have provided unprecedented insights into this complex process.

On the surface of cytotoxic T cells, the TCR (comprising α and β chains) forms a TCR-CD3 complex with CD3 (consisting of γ, δ, ϵ, and ξ components), collectively responsible for specific antigen recognition and signal transduction (75). The advent of high-throughput sequencing and bioinformatics has revolutionized our understanding of tumor-specific antigens. Notably, Schumacher and Schreiber elucidated that endogenous T cells can recognize peptide epitopes displayed on major histocompatibility complexes (MHCs) on the surface of malignant cells, emphasizing their potential as targets for personalized immunotherapies (76). Dustin and Choudhuri further refined the dynamics of TCR engagement with peptide-MHC complexes, revealing the discovery of the immunological synapse and the importance of mechanical forces in T cell activation (77). Their research suggested the possibility of a crossover symmetry plane and regulated symmetry breaking to control the duration of T cell-APC interactions, influencing signal transduction. Furthermore, Hu et al. employed CRISPR-Cas9 screening techniques to identify novel antigen presentation pathways, uncovering non-classical sources of tumor antigens (78). Patskovsky et al. highlighted the role of post-translational modifications in generating tumor-specific epitopes, demonstrating that phosphopeptides can serve as effective T cell targets (79). Additionally, Yan et al. utilized single-cell RNA sequencing to delineate the transcriptional programs of tumor-infiltrating T cells, revealing distinct states of functional impairment and reinvigoration potential (80). Spranger et al. further elucidated the importance of cross-presentation in initiating anti-tumor T cell responses, finding that BATF3-dependent dendritic cells are crucial for priming tumor-specific T cells (81).

These findings collectively underscore the multifaceted nature of T cell recognition in cancer and offer promising avenues for enhancing the efficacy of immunotherapies through targeted manipulation of antigen presentation and T cell priming.

### 3.6 Cancer cell killing

**Figure 2:**
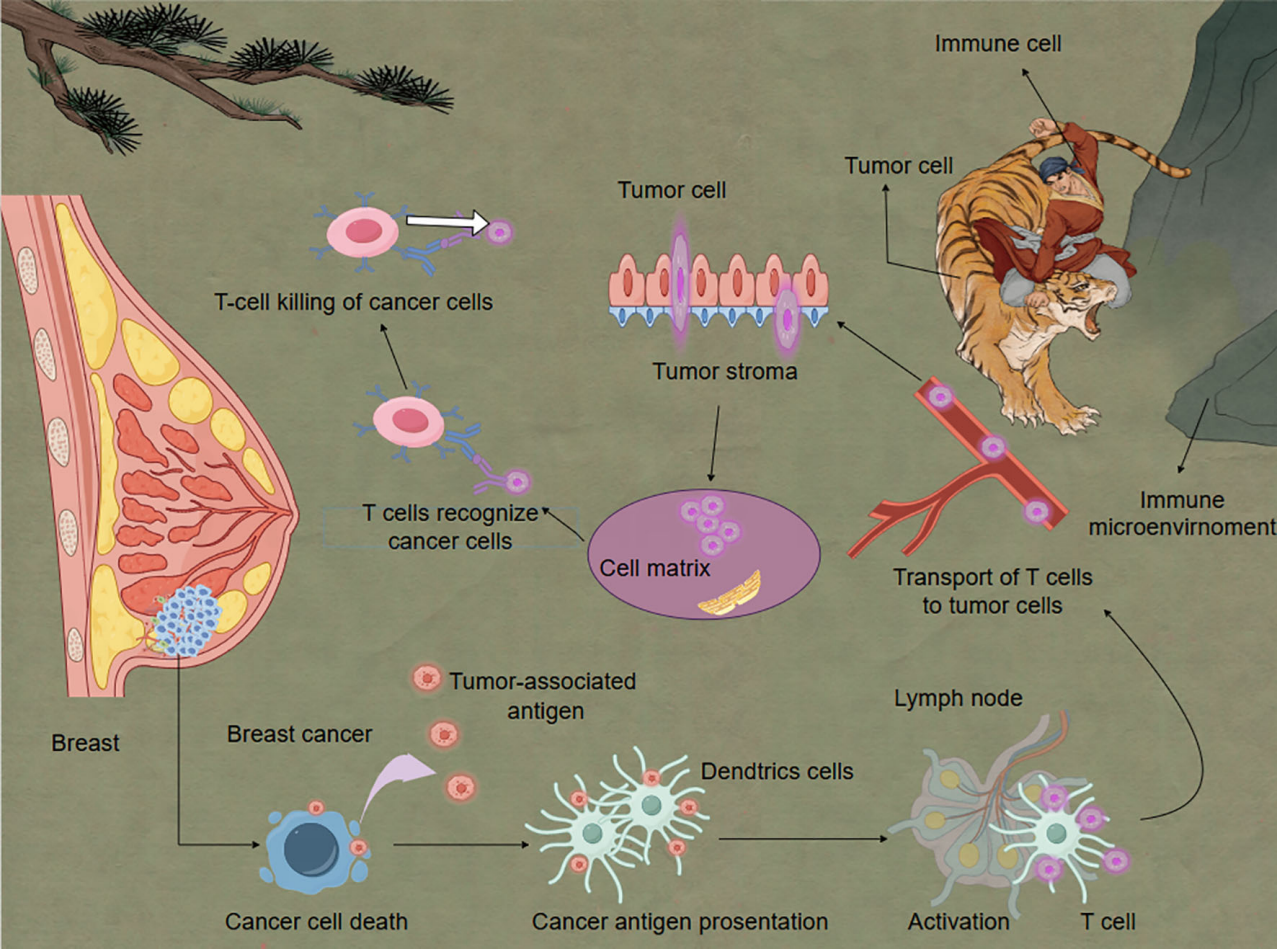
Immune cells eliminate breast cancer cells under the influence of the immune microenvironment. Activated T cells play a critical role in eliminating cancer cells. Dendritic cells serve as essential mediators in T cell activation. When DCs detect antigens derived from cancer cells, they process and present these antigens as peptide fragments on their cell surface via major histocompatibility complexes. These peptide-MHC complexes are subsequently recognized by T cell receptors on naïve T cells, triggering their activation and clonal expansion. This antigen-specific immune response forms the molecular basis for adaptive anti-tumor immunity.

Recent studies have revealed that cancer cell killing involves multiple mechanisms, including cytotoxic T lymphocyte (CTL)-mediated tumor cell killing, NK cell participation in anti-tumor immunity, and regulation of immune cell killing function by the tumor microenvironment (**Figure 2**).

In the killing phase of the cancer-immunity cycle, activated effector T cells recognizing and killing tumor cells is a critical step. Recent research indicates that the process of CTL-mediated tumor cell killing is more complex than previously understood. Xu et al., using real-time imaging techniques, discovered that CTLs form dynamic “killing synapses” upon contact with tumor cells, structures that precisely localize the release of cytotoxic molecules (82). Furthermore, Dunstone et al. found that perforin undergoes conformational changes when forming transmembrane pores, providing new insights for designing perforin-targeting drugs (83). NK cells, as crucial components of the innate immune system, play a key role in tumor immunosurveillance. Recent studies have uncovered new mechanisms of NK cell recognition and killing of tumor cells. Liu et al. discovered that the interaction between the NKG2A receptor on NK cells and HLA-E can inhibit NK cell activity, and blocking this interaction can enhance NK cell anti-tumor effects (84). Additionally, researchers found that metabolic reprogramming in the tumor microenvironment affects NK cell function, offering new directions for designing NK cell metabolic regulation strategies. The tumor microenvironment significantly influences the killing function of immune cells. Wang et al. found that TAMs can suppress CTL effector functions by secreting IL-10 (85). Targeting this mechanism, they developed antibodies against the IL-10 receptor, significantly enhancing CTL anti-tumor activity. Moreover, Qian et al. discovered that prostaglandin E2 (PGE2) secreted by tumor cells can inhibit dendritic cell cross-presentation, thereby affecting CTL activation. Paclitaxel (PTX) can induce immunogenic cell death, and the combination of celecoxib and PTX promoted dendritic cell maturation and elicited T cell-dependent immune responses. Inhibiting PGE2 synthesis can enhance the efficacy of immune checkpoint inhibitors (86).

After killing cancer cells, T cells enter a dysfunctional exhaustion state. T cell exhaustion is also crucial in affecting immune responses. Tex cells have reduced effector functions due to decreased cytokine production, increased production of immunosuppressive enzymes like CXCL13 and CD39, and higher expression of inhibitory receptors such as CTLA4 and LAG3 (87–89). Contrary to previous views, recent studies suggest that Texterm cells can produce effector molecules at the transcriptional level, and are considered key barriers to immunotherapy (90). Sun et al.’s research demonstrated that Stat3 deficiency leads to a significant decrease in the proportion and number of tumor-specific Texterm cells, as well as significantly impaired granzyme B production capacity (91).

## 4 Application of precision hydrogels in breast cancer therapy

### 4.1 Design principles and characteristics of precision hydrogels

**Figure 3:**
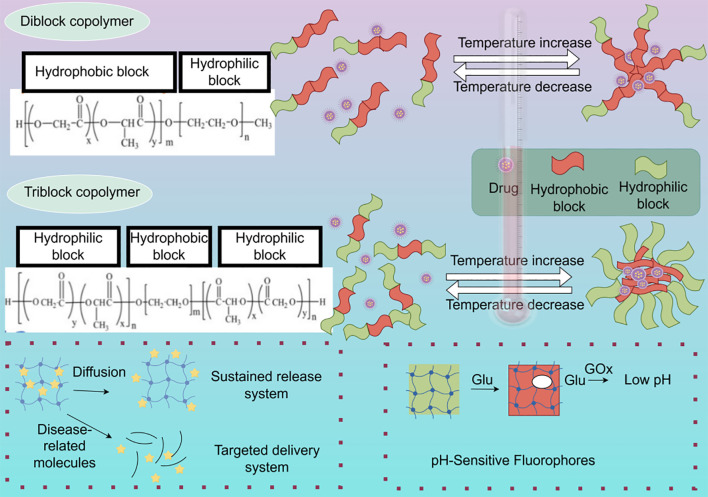
Illustrative overview of sol-gel transition mechanisms and drug loading within copolymer hydrogels and a glucose-responsive system. The diblock copolymer PEG-PLGA forms a gel at higher temperatures via hydrophobic interactions, leading to the encapsulation of hydrophobic drugs within the PLGA core. The triblock copolymer PLGA-PEG-PLGA also undergoes temperature-induced gelation, but exhibits increased hydrophilic PEG junctions and fewer hydrophobic PLGA end groups, influencing gel properties. The diagram further depicts the architecture of a GOx-based hydrogel designed for glucose-sensitive drug release (By figdraw).

With the rapid development of nanotechnology and biomaterials science, precision hydrogels have emerged as a novel drug delivery system with immense potential in breast cancer therapy. Their unique design principles and characteristics enable targeted delivery, controlled release, and multifunctional integration, offering new strategies for precision treatment of breast cancer. The design of precision hydrogels is primarily based on several principles, including biocompatibility and degradability, stimulus-responsiveness, targeting ability, and multifunctional integration (**Figure 3**).

Zheng et al. developed a hydrogel by covalently grafting metformin (Met) onto oxidized hyaluronic acid (OHA) via imine bonds to prepare OHA-Met, subsequently formulating a carboxymethyl chitosan (CMCS)/OHA-Met drug-loaded hydrogel. By modulating the molecular weight and crosslinking degree of the CMCS/OHA-Met20 hydrogel, they achieved effective killing of breast cancer cells and precise degradation of the hydrogel in the breast cancer microenvironment, enabling local injection and pH-responsive intelligent drug delivery to inhibit breast cancer recurrence (92). In addition to stimulus-responsiveness, hydrogels also exhibit multifunctional integration. Recently, researchers reported a hydrogel system integrating diagnostic and therapeutic functions. They co-encapsulated magnetic nanoparticles and chemotherapeutic drugs within the hydrogel, achieving MRI-guided precision therapy (93). Chen et al. manufactured HER2/CD44-targeted hydrogel nanorobots (ALPR) by encapsulating liposome-based nanocomposites (containing survivin and pro-apoptotic peptide drugs) into Herceptin/hyaluronic acid crosslinked nano-hydrogels (94). ALPR can specifically deliver peptides, Herceptin, and siRNA drugs to breast cancer cells, thereby inhibiting cell growth. Moreover, ALPR can suppress breast cancer cell growth through synergistic blockade of HER2-positive cell surface receptors, downregulation of survival genes, and disruption of mitochondrial multi-channels.

**Figure 4:**
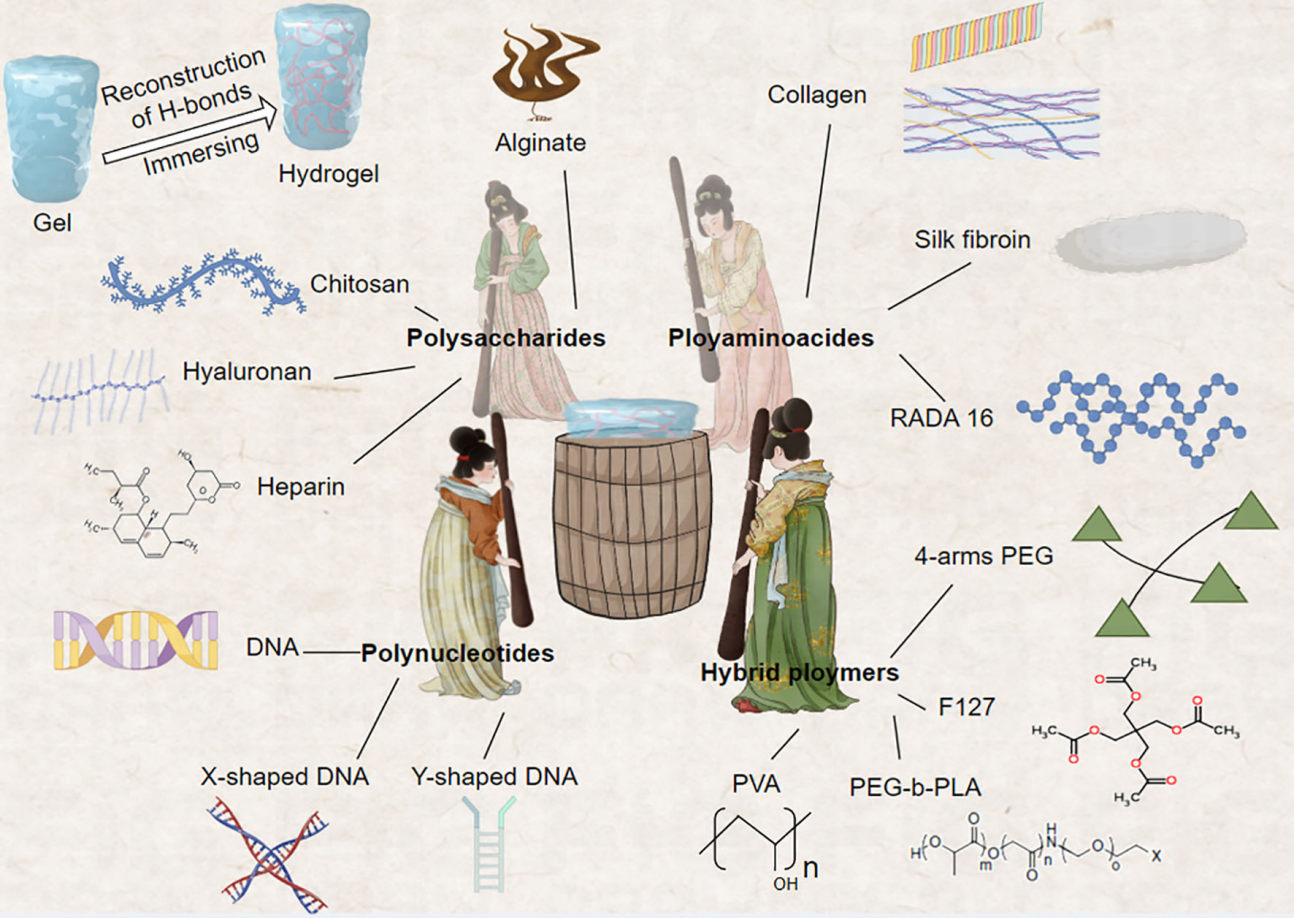
This innovative approach fuses the construction of biopolymer-based hydrogels with the iconic work scene of noble women engaged in silk pounding, as depicted in the renowned Tang Dynasty painting. The natural polymers comprising these hydrogels include gelatin, collagen, hyaluronic acid, alginate, and chitosan, while synthetic polymers encompass poly (N-isopropylacrylamide) and 4-armed polyethylene glycol (4-arms PEG). These diverse material systems can be functionalized using various reversible crosslinking strategies, enabling the creation of dynamic and responsive hydrogel platforms. This biomimetic approach not only draws inspiration from classical Chinese art but also advances the field of bioengineering, offering new possibilities for designing sophisticated hydrogel systems with tailored properties and functions.

Precision hydrogels have demonstrated several excellent characteristics in breast cancer treatment, including high loading capacity, enhanced permeability, and immunomodulatory effects (**Table 2**). Wang et al., utilizing supramolecular self-assembly principles, developed a 1064nm NIR-II light-driven asymmetric hydrogel nanomotor (AHNM) with high motility, loading it with doxorubicin to enhance immunotherapeutic efficacy in cancer patients (99). Zhou et al. synthesized a hydrogel system comprising positively charged DNA nanostructure-based photothermal, highly permeable, and injectable biodegradable nucleic acid hydrogel (DNA-gel) nanoparticles. By designing specific DNA sequences, they achieved multi-stage controlled release of drugs. This precision hydrogel significantly promoted the permeability of drug particles both intracellularly and extracellularly (100). Yin et al. designed a hydrogel system with immunomodulatory functions. Through the combined use of Esketamine and DDP co-loaded in a Poloxamer hydrogel (PDEH), this hydrogel effectively activated anti-tumor immune responses by increasing the number of mature dendritic cells (mDCs) and activated T cells, providing a new strategy for breast cancer immunotherapy (101) (**Figure 4**).

**Table 2:** The structure and function of hydrogels.

Cancer type	Li-Gel hybrid	Liposome			Hydrogel		Study type	Outcome	^Ref^
		Structure	Properties	Payload	Structure	Payload			
Breast cancer	PEGylated liposomal Dox loaded hydrogel	DPPC, Chol, DSPE-PEG2000	120.5 ± 1.9 nm	DOX	F-GelMA	–	*In vitro* (drug release)	Gridline-type patches were the best for DOX sustained release Longer UV exposure (2 min) increased cross linking density and delayed DOX release. An implantable Li-Gel hybrid system	(95)
	DOX and liposomal Cur loaded hydrogel	PC, Chol	268.73 ± 3.19 nm/ -41.30 ± 1.01 mV	Cur	Chitosan	DOX	*In vitro* (MCF-7 cells)	An injectable thermo- and pH-responsive system - Inhibited cancer cell growth Initial rapid release of drugs during first 24 h and their sustained release up to 120 h at acidic condition	(96)
	PEIGel	PEI, PVA and Mg2+	100–300 nm	DOX	–	DOX	*In vitro* (α-PDL1 release)	PEIGel encapsulating α-PDL1 exhibits synergistic effects resulting in elimination of primary tumors and remote metastases and prevention of tumor relapse after surgical resection.	(97)
	Liposomal Cur loaded hydrogel	DMPG, DMPC, Chol	100–300 nm	Cur	SF	–	*In vitro* (MDA-MB-231 cells)	A potential sealant to eradicate remaining malignant cells after tumor resection Liposomes induced SF rapid gelation Inhibited cancer cell growth Burst Cur release during first 24 h and subsequent gradual release until 7 days	(98)

As a novel drug delivery system, precision hydrogels offer new opportunities for precise breast cancer treatment through their unique design principles and excellent characteristics. In the future, by further optimizing hydrogel performance and strengthening preclinical evaluation and translational research, precision hydrogels are poised to become an important tool in breast cancer therapy.

### 4.2 Targeted delivery of immunomodulators

With the rapid development of cancer immunotherapy, precision hydrogels not only enhance the targeting and efficacy of immunomodulators but also reduce systemic toxicity, paving new avenues for breast cancer immunotherapy.

Immune checkpoint inhibitors (such as PD-1/PD-L1 antibodies) have shown promising results in breast cancer treatment, but systemic administration may lead to severe immune-related adverse events. Ye et al. prepared black phosphorus quantum dot nanovesicles coated with surgically resected tumor cell membranes (BPQD-CCNV) and loaded them into a thermosensitive hydrogel containing GM-CSF and LPS. Subcutaneous injection of this hydrogel resulted in sustained release of GM-CSF, effectively recruiting dendritic cells to capture tumor antigens. This system leveraged the weakly acidic characteristics of the breast cancer microenvironment to trigger antibody release, significantly enhancing antibody accumulation at the tumor site while reducing systemic exposure (102). Preclinical studies demonstrated that this strategy not only enhanced anti-tumor immune responses but also significantly reduced the incidence of immune-related adverse events. Chao et al. reported an alginate-based hydrogel system where soluble polysaccharides rapidly transform into hydrogels in the presence of endogenous Ca2+, immobilizing 131I-Cat within the tumor (103). By precisely controlling the release kinetics of two antibodies, this system achieved synergistic immune activation effects, exhibiting excellent anti-tumor activity in triple-negative breast cancer models.

Cytokines and immune adjuvants play crucial roles in activating and modulating anti-tumor immune responses, but systemic administration often leads to severe toxicity. Hori et al. designed a multi-layered hydrogel system for delivering IL-2 and CpG oligonucleotides (104). This system achieved sequential release of IL-2 and CpG by regulating the degradation rates of different layers, effectively mimicking the natural immune activation process. In HER2-positive breast cancer models, this strategy significantly enhanced the anti-tumor activity of NK cells and CD8+ T cells. Giang et al. reported a DNA nanostructure-based hydrogel system for co-delivering GM-CSF and tumor antigens (105). The sustained degradation of the microporous hydrogel network maintained the release of DNA vaccines. Through precise design of DNA sequences, this system could respond to adenosine concentrations in the tumor microenvironment, achieving intelligent release. This strategy not only enhanced the recruitment and activation of dendritic cells but also significantly improved antigen-specific T cell responses.

While CAR-T cell therapy has achieved breakthrough progress in hematological malignancies, its efficacy in solid tumors such as breast cancer remains limited. Researchers have developed an injectable hydrogel system for local delivery of CAR-T cells. This system not only provides a suitable microenvironment for CAR-T cells but also continuously releases cytokines such as IL-15, significantly improving the survival and proliferation of CAR-T cells at the tumor site (106). In refractory breast cancer models, this strategy significantly enhanced the anti-tumor effect of CAR-T cells. Regulatory T cells (Tregs) play a crucial immunosuppressive role in the breast cancer microenvironment. Researchers designed a hydrogel system for targeted delivery of Treg inhibitors. By modifying the hydrogel surface with CCR4 ligands, this system can specifically target Tregs. In breast cancer models, this strategy significantly reduced the number of tumor-infiltrating Tregs and enhanced the anti-tumor activity of effector T cells (107, 108). Personalized tumor vaccines represent a frontier in precision immunotherapy for breast cancer. Zeng et al. reported an mRNA-based hydrogel vaccine delivery system. This system effectively protects mRNA from degradation while achieving slow release, significantly enhancing antigen-specific T cell responses (109). In preclinical breast cancer models, this strategy induced potent anti-tumor immune responses. To achieve multiple immunomodulation, researchers developed a multifunctional integrated hydrogel system. This system simultaneously encapsulates immune checkpoint inhibitors, cytokines, and photosensitizers. This synergistic strategy demonstrated significant anti-tumor effects and inhibition of distant metastases in breast cancer models.

Precision hydrogels have demonstrated immense potential in the targeted delivery of immunomodulators, offering novel strategies for breast cancer immunotherapy. Through precise design and multifunctional integration, these systems can achieve targeted delivery, controlled release, and synergistic effects of immunomodulators, significantly enhancing therapeutic efficacy while reducing toxicity. In the future, with further advancements in biomaterials science and immunology, precision hydrogels are poised to become a crucial platform for personalized immunotherapy in breast cancer treatment.

### 4.3 Mimicking the tumor microenvironment to enhance t cell function

With the advancement of cancer immunotherapy research, there is growing recognition of the critical role the tumor microenvironment (TME) plays in modulating T cell function. Precision hydrogels, as designable three-dimensional microenvironments, offer a unique platform for mimicking and manipulating the breast cancer TME, thereby enhancing the anti-tumor function of T cells. This innovative strategy not only contributes to a deeper understanding of T cell interactions with the TME but also provides an important tool for developing novel immunotherapeutic approaches.

The physical characteristics of the breast cancer TME, such as matrix stiffness and topological structure, significantly influence T cell infiltration and function. Researchers developed a hydrogel system with tunable stiffness, mimicking breast cancer tissue hardness at different stages by precisely controlling crosslinking density. They found that hydrogels of intermediate stiffness were most conducive to T cell migration and activation. Based on this finding, they designed a stiffness gradient hydrogel that significantly enhanced T cell infiltration toward the tumor center. Talin-low MDA-MB-231 breast cancer cells migrated along stiffness gradients through durotaxis or softotaxis mechanisms, reaching optimal stiffness zones and tending to generate maximum traction on substrates of intermediate stiffness (110). Furthermore, researchers reported a DNA nanotechnology-based hydrogel system capable of dynamically regulating pore structure. Through the design of specific DNA sequences, this system could respond to tumor-secreted proteases, adjusting network structure in real-time to promote deep T cell penetration. In DNA-crosslinked hydrogels where polymer long chains are crosslinked by DNA strands, substrate stiffness can be altered without environmental stimuli (111). In triple-negative breast cancer models, this strategy significantly enhanced the anti-tumor effect of CAR-T cells. Biochemical signals in the TME are crucial for T cell activation and function. Berger et al. designed a multi-layered hydrogel system to simulate cytokine gradients in the breast cancer TME. By encapsulating factors such as IL-2, IL-15, and CXCL10 in different layers, this system could precisely control the spatiotemporal distribution of these factors, effectively simulating the T cell activation process in the TME (112). *In vivo* studies demonstrated that this strategy significantly enhanced the proliferation and effector functions of tumor-infiltrating lymphocytes (TILs).

Lo et al. reported the development of a novel injectable hydrogel composed of maleimide-modified γ-polyglutamic acid (γ-PGA-MA) and thiol-terminated 4-arm polyethylene glycol (4-arm PEG-SH), capable of dynamically presenting tumor antigens. Through the design of specific peptide sequences, this system could respond to proteases in the TME, releasing new tumor antigen epitopes in real-time. This dynamic antigen presentation strategy significantly enhanced T cell activation and persistence, demonstrating excellent anti-tumor effects in HER2-positive breast cancer models (113). The metabolic characteristics of the TME significantly impact T cell function. Chen et al. developed a hydrogel system with metabolic regulatory functions. By encapsulating glucose transporter inhibitors and lactate dehydrogenase inhibitors, this system could locally regulate the metabolic state of the TME, reducing lactate accumulation and providing a more favorable metabolic environment for T cells (114). In refractory breast cancer models, researchers significantly enhanced the function and persistence of TILs. The breast cancer TME often exhibits strong immunosuppressive properties, affecting T cell function. Xiao et al. designed a hydrogel system mimicking the immunosuppressive TME by integrating inhibitory factors such as PD-L1, TGF-β, and IDO, creating a highly immunosuppressive microenvironment. Using this system, they screened for highly resistant T cell subsets and further enhanced their function through gene editing (115). These “TME-trained” T cells demonstrated significantly enhanced anti-tumor activity *in vivo*.

Precision hydrogels not only serve to directly enhance T cell function but also provide crucial tools for in-depth investigation of T cell interactions with the TME. In the future, as biomaterials science, microfluidic technology, and immunology continue to converge, TME simulation systems based on precision hydrogels are poised to become a key driving force in advancing breast cancer immunotherapy. These innovative platforms offer unprecedented opportunities to unravel the complexities of the TME and develop more effective, personalized immunotherapeutic strategies.

### 4.4 Controlled release of tumor antigens and adjuvants

With the continuous advancement of tumor vaccine strategies, precise control over the release of tumor antigens and adjuvants has become crucial for enhancing vaccine efficacy. Precision hydrogels, as designable delivery systems, offer a unique platform to achieve this goal. By accurately modulating the release kinetics of antigens and adjuvants, these systems not only enhance the activation of antigen-presenting cells (APCs) but also induce more potent and sustained anti-tumor immune responses, bringing new opportunities for breast cancer immunotherapy.

Sequential release strategies mimicking natural immune activation processes have been proven to significantly enhance vaccine efficacy. Shih et al. developed a multi-layered hydrogel-based delivery system for controlled release of GM-CSF, CpG, and tumor antigens. By precisely designing the degradation rate of each layer, this system first releases GM-CSF to recruit APCs, followed by CpG to activate APCs, and finally releases tumor antigens (116). In triple-negative breast cancer models, this sequential release strategy significantly enhanced antigen-specific T cell responses and inhibited tumor growth. Researchers reported a DNA nanotechnology-based hydrogel system capable of achieving more refined multi-step release. By designing DNA nanostructures with varying stabilities, this system can release multiple adjuvants and antigens according to a preset program, simulating complex immune activation cascades (117). This strategy induced potent anti-tumor immune memory in HER2-positive breast cancer models, significantly prolonging mouse survival. Researchers developed a multifunctional cationic nano-hydrogel self-adjuvant anti-tumor vaccine loaded with CpG as an immunostimulant for *in situ* delivery. The complexation of oligonucleotides with CpG enhanced toll-like receptor (TLR) stimulated T cell proliferation and rapid immune activation. This system not only locally releases TLR agonists and anti-CD40 antibodies but also captures and processes released tumor antigens. In metastatic breast cancer models, this strategy induced potent systemic anti-tumor immune responses and inhibited distant metastases (118, 119).

Precision hydrogels demonstrate unique advantages in controlling the release of tumor antigens and adjuvants. Through strategies such as sequential release, responsive release, and sustained release, these systems can accurately simulate and enhance the immune activation process. By precisely orchestrating the spatiotemporal presentation of immunomodulatory factors, precision hydrogels offer a powerful platform for optimizing cancer vaccine efficacy and advancing breast cancer immunotherapy.

## 5 Novel strategies for enhancing the cancer-immunity cycle using precision hydrogels

### 5.1 Multifunctional hydrogel systems

The cancer-immunity cycle is a complex, multi-step process encompassing critical stages such as antigen capture and presentation, T cell activation and infiltration, and tumor recognition and killing (120–122). Multifunctional hydrogel systems integrate various functional modules through synergistic immune modulation, regulation of multiple immune checkpoints, and remodeling of the tumor microenvironment. These systems provide a unique platform for simultaneously regulating multiple steps of the cancer-immunity cycle.

Efficient antigen capture and presentation are crucial for initiating the cancer-immunity cycle. Recently, researchers have developed a hydrogel system with *in situ* antigen capture and processing capabilities. This system integrates multiple functional modules: pH-responsive nanoparticles for capturing and protecting antigens from tumor lysates; protease-sensitive peptide linkers for controlled antigen release; and TLR agonists for activating dendritic cells (DCs) (123). In triple-negative breast cancer models, this multifunctional system significantly enhanced antigen capture efficiency and DC activation, inducing potent anti-tumor immune responses. Addressing the synergistic effects of multiple immune checkpoints in breast cancer, researchers designed a multi-checkpoint regulatory hydrogel. This system incorporates various functions: anti-PD-1 antibodies to block the PD-1/PD-L1 pathway, anti-CTLA-4 antibodies to enhance T cell activation, and anti-LAG-3 antibodies to restore T cell function (124). By optimizing the release ratios and timing of different antibodies, this system achieved synergistic blockade of multiple immune checkpoints.

In recent years, researchers have developed an injectable, near-infrared laser-responsive hydrogel loaded with multiple nanotherapeutic agents for post-tumor resection immunoactivation. This hydrogel system simultaneously reshapes the tumor microenvironment and activates immune responses. Through synergistic treatment combining photodynamic therapy, chemotherapy, and immunostimulants, it enhances the cancer-immunity cycle. The system integrates the following functional modules: a) CCL2 inhibitors to reduce infiltration of immunosuppressive macrophages; b) TGF-β inhibitors to reverse T cell functional suppression; c) IL-12 to enhance T cell and NK cell activation; d) photosensitizers to induce immunogenic cell death (ICD) (125). Furthermore, the experiment confirmed that iGEL could induce tumor cell killing, polarization of tumor-associated macrophages toward the M1 phenotype, ICD, dendritic cell maturation, and enhanced antigen presentation capability. In breast cancer models, this multifunctional system not only improved the local immune microenvironment but also increased tumor antigen release through ICD, achieving synergistic regulation of multiple stages in the cancer-immunity cycle.

Multifunctional hydrogel systems provide a powerful and flexible platform for enhancing the breast cancer immunity cycle. By integrating multiple functional modules, these systems can simultaneously regulate several critical stages of the cancer-immunity cycle, achieving synergistic enhancement. This approach offers unprecedented opportunities for comprehensive modulation of anti-tumor immune responses, potentially leading to more effective and personalized immunotherapeutic strategies in breast cancer treatment.

### 5.2 Intelligent responsive hydrogels

**Figure 5:**
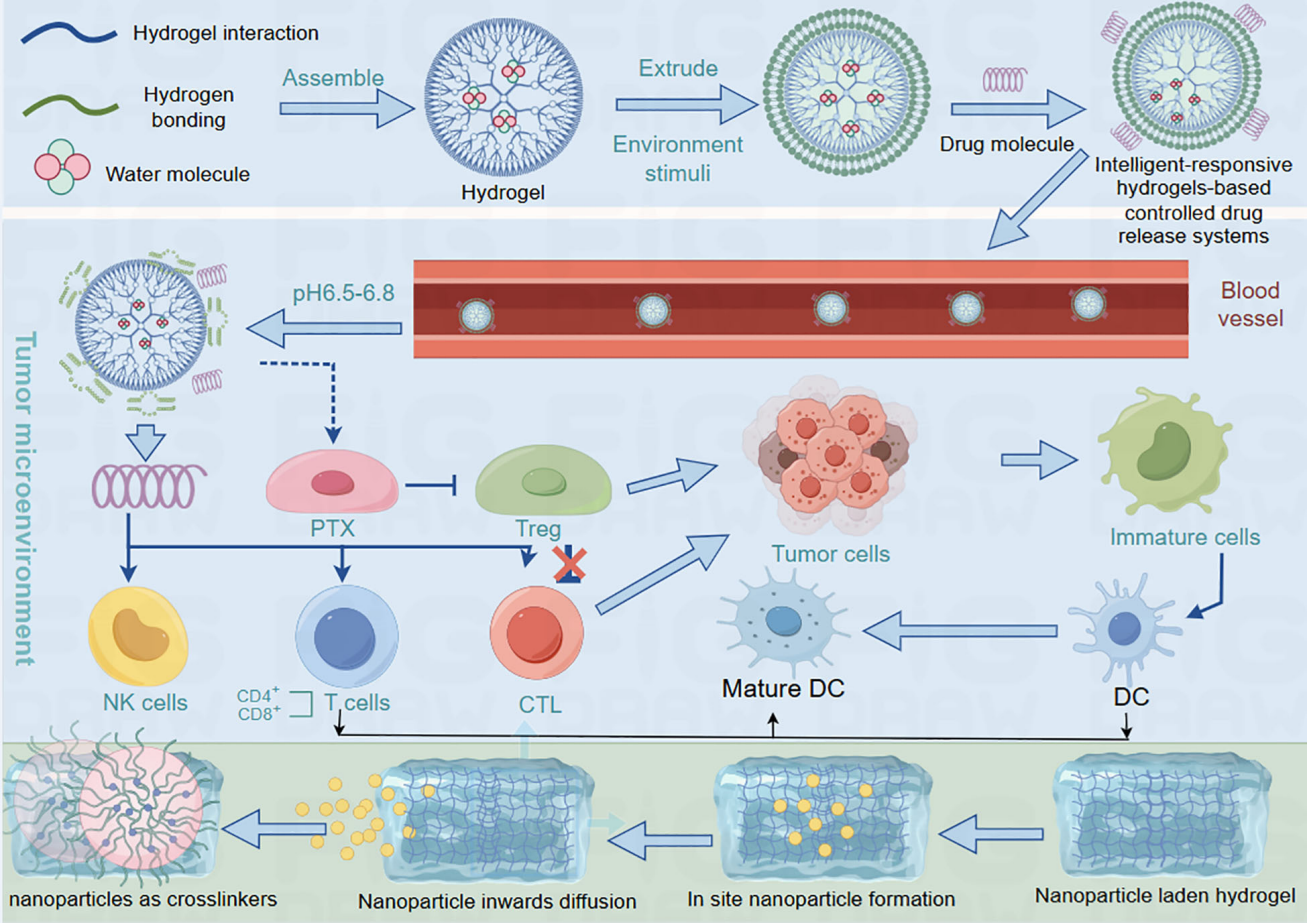
The process of hydrogel releasing drugs into the blood. Hydrogels absorb water to release drugs into the bloodstream, which then spread throughout the body. Their interactions with the immune system are regulated by the surrounding biological environment. When combined with nanoparticles through diffusion-based integration, these nanocomposite hydrogels form advanced drug delivery systems. The nanoparticle-hydrogel combination allows more precise control of drug release and biological interactions, showing particular promise for improving cancer therapies.

With the deepening understanding of the complexity of the breast cancer immune microenvironment, intelligent responsive hydrogel systems, through their responsiveness to various stimuli such as pH, enzymes, temperature, and redox conditions, can achieve targeted drug delivery, dynamic regulation of immune cells, and remodeling of the tumor microenvironment. These advanced systems offer unprecedented precision in modulating the intricate interplay between cancer cells and the immune system, potentially revolutionizing breast cancer immunotherapy strategies (**Figure 5**).

The altered pH in pathological sites provides a foundation for developing pH-responsive hydrogels. Edirisinghe et al. designed a pH-responsive hydrogel that leverages the mildly acidic microenvironment (pH 6.5-6.8) of breast cancer lesions to trigger structural changes and drug release (126, 127). This system remains stable at physiological pH but rapidly swells and releases drugs in the acidic tumor environment. This design significantly enhanced chemotherapeutic drug accumulation at tumor sites while reducing systemic toxicity. *In vivo* studies demonstrated that this strategy markedly increased anti-PD-1 antibody accumulation in tumors while minimizing systemic toxicity. The overexpression of specific enzymes in the breast cancer microenvironment offers opportunities for developing enzyme-responsive hydrogels. Researchers designed a hydrogel system sensitive to matrix metalloproteinases (MMPs). This system, linked by MMP-cleavable peptide sequences, undergoes specific degradation at tumor sites. This strategy not only achieved targeted drug release but also promoted immune cell infiltration through dynamic adjustment of the network structure. Researchers reported an advanced multi-enzyme responsive system integrating the following features: a) MMP-responsive backbone for controlling overall degradation; b) Cathepsin B-responsive linkers for controlling antigen release; c) γ-glutamyl transpeptidase-responsive prodrugs for activating immunomodulators (128). This sophisticated design achieved precise regulation of multiple stages in the cancer-immunity cycle, demonstrating excellent anti-tumor efficacy in HER2-positive breast cancer models.

In recent years, research on intelligent responsive hydrogels has flourished. Researchers have utilized local heating or near-infrared light irradiation to trigger phase transitions in temperature-responsive hydrogels, achieving precise control of drug release. In these smart responsive hydrogel drug delivery systems, certain segments of the polymer chains undergo a “soluble-insoluble” phase transition process within a specific temperature range. This phase transition generally occurs in two scenarios. The first involves a lower critical solution transition temperature (LCST). The second involves an upper critical solution transition temperature (UCST), where the polymer chains typically contain polar groups capable of association, such as poly (sulfobetaine methacrylate) (PSPE), a zwitterionic polyelectrolyte. When the temperature rises to a certain point, the thermoresponsive segment of the polymer chain transitions from an insoluble (phase-separated) state to a soluble state (129–131). Hauck et al. developed a temperature-sensitive hydrogel system based on poly (N-isopropylacrylamide) (PNIPAM). This system remains stable at body temperature but rapidly contracts to release encapsulated CAR-T cells upon local heating (42℃). This strategy significantly enhanced CAR-T cell infiltration and persistence in solid tumors (132).

Hao et al. reported an intelligent hydrogel system integrating photothermal therapy and immunomodulation. The system includes gold nanorods for photothermal conversion under near-infrared light excitation, a temperature-responsive hydrogel backbone for controlled drug release, and immune checkpoint inhibitors and IL-2 (133). In breast cancer models, this system induced immunogenic cell death through photothermal therapy while releasing immunomodulators, achieving synergistic activation of local and systemic anti-tumor immune responses.

The abnormal redox state in the breast cancer microenvironment provides a basis for developing corresponding intelligent hydrogels. Zhu et al. designed a glutathione (GSH)-sensitive hydrogel system. They encapsulated SS and O-CMC in the hydrogel, which was cross-linked through disulfide bonds and rapidly degraded in the high GSH environment of tumors. This strategy not only achieved targeted release of antigens and adjuvants but also altered the tumor’s redox state by consuming GSH, effectively killing breast cancer cells without affecting non-cancer cells and enhancing T cell effector functions (134). The latest research is developing intelligent feedback-type hydrogel systems with self-regulatory capabilities. Researchers have designed a dynamic hydrogel system based on aptamers. This system can sense the concentration of specific cytokines and adjust drug release rates accordingly. This adaptive strategy holds promise for real-time precise regulation of immune responses, offering new possibilities for personalized breast cancer immunotherapy.

### 5.3 Nanohydrogel technology

Nanohydrogels, as an emerging class of nanomaterials, enable precise modulation of multiple stages in the cancer-immunity cycle. Combining the high targeting capability of nanoparticles with the biocompatibility of hydrogels, nanohydrogels achieve efficient targeted delivery, multifunctional integration, intelligent responsiveness, and biomimicry. These features provide significant advantages in enhancing the breast cancer immunity cycle. The unique properties of nanohydrogels offer unprecedented opportunities for developing advanced immunotherapeutic strategies, potentially revolutionizing breast cancer treatment approaches.

The nanoscale characteristics of nanohydrogels enable efficient penetration through tumor vasculature and interstitial spaces, achieving precise targeting. Zhang et al. developed a nanohydrogel system based on polyethylene glycol-polylactic acid (PEG-PLA) for delivering STING agonists. This system accumulated in breast cancer tissues via the EPR effect, significantly enhancing type I interferon production (135). *In vivo* studies demonstrated that this strategy not only activated dendritic cells but also promoted CD8+ T cell infiltration, effectively inhibiting tumor growth. Li et al. reported a more advanced active-targeting nanohydrogel system. By modifying the surface with HER2-targeting peptides, this system could specifically recognize HER2-positive breast cancer cells. This precise targeting strategy significantly increased anti-PD-1 antibody accumulation at tumor sites while reducing systemic toxicity (108). Nanohydrogels provide an ideal platform for integrating multiple functional modules. Researchers designed a multifunctional nanohydrogel system incorporating the following components: a) a core-shell structure for separately encapsulating tumor antigens and TLR agonists; b) a pH-responsive outer layer for triggering content release; c) surface-modified PD-L1 antibodies (136). This design achieved synergistic effects of antigen presentation, immune activation, and immune checkpoint blockade. In triple-negative breast cancer models, the system induced potent anti-tumor immune responses, significantly prolonging mouse survival.

Through intelligent responsive materials, nanohydrogels can achieve precise responses to the breast cancer microenvironment. Huang et al. reported a multi-responsive nanohydrogel system with the following characteristics: a) MMP2-responsive linkers for triggering drug release; b) pH-sensitive polymer backbone for enhancing intracellular escape; c) redox-responsive crosslinkers for controlling overall degradation (137). In the complex breast cancer microenvironment, this system can sequentially respond to different stimuli, achieving precisely controlled drug release and nanoparticle degradation. *In vivo* studies demonstrated that this strategy significantly enhanced the delivery efficiency and immunogenicity of mRNA vaccines. Nanohydrogels mimicking biological structures exhibit unique advantages. Patwardhan et al. developed a nanohydrogel system mimicking extracellular vesicles. By incorporating cell membrane components and specific proteins, this system not only possessed biocompatibility but also evaded immune clearance. This biomimetic strategy significantly increased the half-life of nanohydrogels in circulation, enhancing their accumulation in breast cancer tissues (138). *In situ* formation techniques offer new possibilities for nanohydrogel applications. Researchers reported an *in situ* forming nanohydrogel system based on supramolecular self-assembly. This system could rapidly respond to specific signals (such as ATP) in the tumor microenvironment after injection, spontaneously forming nanohydrogel networks (139). This strategy not only achieved sustained drug release but also enhanced the retention of immune cells at tumor sites through a physical barrier effect.

Real-time monitoring of the *in vivo* distribution and function of nanohydrogels is crucial for optimizing therapeutic strategies. In addition to the aforementioned studies, researchers have designed a multimodal imaging nanohydrogel system integrating various features: superparamagnetic iron oxide nanoparticles for MRI imaging, near-infrared fluorescent dyes for optical imaging, and 64Cu labeling for PET imaging (140–142). This system enables precise tracking of nanohydrogels throughout the body and within the tumor microenvironment, providing essential data for developing personalized treatment protocols. Shyam Sunder et al. (9) designed an mRNA-based nanohydrogel vaccine system. By precisely controlling mRNA release kinetics and intracellular delivery, this system significantly enhanced the duration and intensity of antigen expression (143).

These studies demonstrate that nanohydrogel technology offers novel strategies for enhancing cell therapy efficacy and developing new cancer vaccines. Future in-depth research in this field holds promise for benefiting breast cancer patients. The integration of advanced nanohydrogel systems with emerging immunotherapeutic approaches may lead to breakthrough treatments, potentially revolutionizing breast cancer management and improving patient outcomes.

### 5.4 Synergistic effects of hydrogels with other immunotherapies

From enhancing the efficacy of ICIs to augmenting cell therapies, from optimizing vaccine strategies to remodeling the tumor microenvironment, hydrogel-mediated synergistic immunotherapies demonstrate immense potential.

Immune checkpoint inhibitors (ICIs) have shown promising results in breast cancer treatment, but response rates remain limited. Teoh et al. developed a hydrogel system for co-delivering anti-PD-1 antibodies and IL-2. This system achieved sustained administration of ICIs and pulsatile release of IL-2 through controlled release, effectively mimicking natural immune activation processes (144). In triple-negative breast cancer models, this synergistic strategy significantly enhanced T cell infiltration and activation, overcoming resistance to ICI monotherapy. By precisely controlling the release kinetics of different inhibitors, the system achieved synergistic blockade of multiple immunosuppressive pathways. Novel tumor vaccines represent a frontier in breast cancer immunotherapy, and hydrogel technology provides an innovative platform for enhancing vaccine potency. Researchers reported a ternary hydrogel composed of polyethyleneimine (PEI), polyvinyl alcohol (PVA), and magnesium ions (a stimulant of adaptive immune responses). They found that PEI hydrogel (PEIGel) encapsulating immune checkpoint blockade (ICB) inhibitors demonstrated synergistic effects with anti-PD-L1 antibodies, eliminating primary tumors and distant metastases, thereby preventing tumor recurrence after surgical resection (97).

Cheng et al. reported an mRNA-based hydrogel vaccine system integrating the following functions: a) mRNA-encoded personalized neoantigens, b) TLR7/8 agonists, and c) STING agonists (145). This design achieved synergistic effects of antigen expression and innate immune activation. In a clinical trial involving metastatic breast cancer patients, this system induced potent neoantigen-specific T cell responses and showed encouraging clinical outcomes when combined with PD-1 inhibitors (102). Radiotherapy can increase tumor antigen release but may also induce immunosuppression. Researchers developed a radio-immunotherapy synergistic hydrogel system containing the following components: a) radiosensitizers, b) CCR2 inhibitors (suppressing immunosuppressive macrophage infiltration), and c) CD40 agonists (promoting antigen presentation) (146, 147). In breast cancer models, this strategy not only enhanced the local effects of radiotherapy but also induced significant distant anti-tumor effects through immune activation.

Considering the heterogeneity of breast cancer, multimodal immunotherapy strategies demonstrate greater advantages. In refractory breast cancer models, this multimodal approach achieves significant synergistic anti-tumor effects by inducing immunogenic cell death through photodynamic therapy, while simultaneously enhancing immune cell function and blocking immunosuppressive pathways. This integrated strategy addresses multiple aspects of tumor-immune interactions, potentially overcoming the limitations of single-modality treatments and offering a more comprehensive approach to combat the complex nature of breast cancer.

## 6 Challenges and future directions

### 6.1 Biocompatibility and safety concerns

Despite hydrogel materials being generally considered biocompatible, they still face numerous challenges in complex biological environments, especially during long-term applications. In-depth understanding and addressing these issues are crucial for advancing precision hydrogel technology toward clinical translation.

The degradation products of hydrogels and their metabolic processes are key to safety assessments. In recent years, researchers have systematically studied the degradation kinetics and metabolic pathways of different types of hydrogels in breast tissue. They found that degradation products of certain synthetic polymers might accumulate locally, inducing chronic inflammatory responses (148). To address this issue, they developed a natural polysaccharide-based hydrogel system whose degradation products can be fully metabolized, significantly reducing the risk of long-term inflammation. Researchers reported an intelligent degradable hydrogel that responds to specific enzymes in the tumor microenvironment for targeted degradation (149, 150). This design not only improved the precision of drug delivery but also reduced potential long-term toxicity by accelerating material clearance. However, they also noted that rapid degradation might lead to burst drug release, necessitating a balance between degradation rate and therapeutic efficacy.

Long-term implanted hydrogel systems face greater biocompatibility challenges. Ren et al. investigated the *in vitro* degradation of HA-PEG hydrogels by incubating 7% (w/v) hydrogels in blank PBS and PBS containing hyaluronidase. The addition of hyaluronidase to the medium accelerated the degradation rate of the hydrogel due to the cleavage of the HA backbone. In the presence of hyaluronidase, HA-PEG hydrogels rapidly degraded within 11 days and exhibited good *in vitro* biocompatibility (151). Hydrogel-induced immunogenicity and inflammatory responses are another important safety concern. Kopyeva et al. conducted a systematic immunotoxicity assessment of various hydrogel materials. They found that certain synthetic polymers might cause adverse reactions by activating the complement system or stimulating macrophages to secrete inflammatory factors. To address this issue, they developed a “stealth” hydrogel based on PEGylation technology, significantly reducing the immunogenicity of the material (152). This design not only lowered the inflammatory response of the material itself but also inhibited tumor-associated chronic inflammation, providing a new approach to solving the immunocompatibility problems of hydrogels.

Considering the heterogeneity of breast cancer patients, individualized safety assessments are becoming increasingly important. Pospelov et al. developed a patient-derived three-dimensional breast cancer organoid model to evaluate the personalized biocompatibility of hydrogel systems (153). This approach can predict potential adverse reactions of specific patients to hydrogel materials, providing a new tool for developing individualized treatment plans and understanding hydrogel biocompatibility (154). Researchers reported a real-time imaging technique based on multiphoton microscopy and Raman spectroscopy, capable of monitoring the degradation process of hydrogels and their interactions with surrounding tissues at the *in vivo* level (155, 156). This technology provides crucial support for optimizing hydrogel design and assessing long-term safety. Through innovations in material design, advanced evaluation techniques, and personalized strategies, we are poised to develop safer and more effective hydrogel systems. These advancements not only address current biocompatibility challenges but also pave the way for the next generation of precision hydrogel-based therapies in breast cancer treatment.

### 6.2 Large-scale production and quality control

Large-scale production and quality control of hydrogels have become key challenges in advancing clinical translation. Addressing these issues requires not only innovative engineering techniques but also the establishment of rigorous standardized processes to ensure product consistency, safety, and efficacy.

Chen et al. investigated the variability of hydrogel network structures in large-scale production. They found that differences in cross-linking density and pore distribution between batches could significantly affect drug release kinetics. To address this issue, they developed a continuous production method based on microfluidic technology, which significantly improved batch-to-batch consistency (157). Sabhachandani et al. reported an automated platform for producing multifunctional immunomodulatory hydrogels. This system integrates precise metering, multi-step reactions, and *in-situ* analysis functions, achieving high-throughput preparation of complex hydrogel systems (158). However, they also pointed out that certain bioactive molecules might be inactivated during large-scale production, necessitating optimized protection strategies. Jiménez et al. systematically evaluated contamination risks in hydrogel production processes. They found that traditional sterilization methods such as high temperature and irradiation could affect the structure and function of hydrogels. To address this, they developed a sterile processing technique based on supercritical CO2, which achieved efficient sterilization while maintaining hydrogel performance (159). Researchers analyzed the cost structure of precision hydrogels from laboratory to industrialization. They pointed out that certain high-value components (such as specific antibodies or growth factors) might lead to cost surges in large-scale production. To address this issue, they proposed an *in-situ* functionalization strategy based on bioorthogonal chemistry, significantly reducing production costs.

Saha et al. developed a comprehensive characterization method to evaluate key quality attributes of hydrogels. This method combines techniques such as dynamic light scattering, atomic force microscopy, and small-angle X-ray scattering to comprehensively analyze the particle size distribution, network structure, and mechanical properties of hydrogels (160). They emphasized that establishing standardized characterization processes is crucial for ensuring batch-to-batch consistency (161). Kheiri et al. reported a high-throughput bioactivity screening platform based on microfluidic chips. This system can evaluate drug release kinetics and immunomodulatory functions of hydrogels at the organoid level, providing a new tool for real-time quality control in large-scale production processes (162). Researchers developed an ultra-high performance liquid chromatography-mass spectrometry method for detecting trace impurities and degradation products in hydrogels. This method achieves ppb-level detection sensitivity, providing crucial support for ensuring product purity and safety (163). Furthermore, researchers systematically studied the effects of different storage conditions on hydrogel performance. They found that certain functionalized hydrogels may undergo structural changes or active component degradation during long-term storage. Based on these findings, they developed an intelligent packaging system capable of real-time monitoring of product status and regulating storage environments (164).

Large-scale production and quality control of precision hydrogels in breast cancer treatment face numerous challenges but also harbor immense opportunities. Through innovative engineering technologies, advanced analytical methods, and intelligent manufacturing strategies, we are poised to overcome current technical bottlenecks and achieve scalable, standardized production. These advancements not only address immediate challenges in hydrogel manufacturing but also pave the way for the broader clinical application of precision hydrogel-based therapies in breast cancer management.

### 6.3 Development of personalized treatment strategies

As our understanding of breast cancer heterogeneity deepens, personalized treatment has become a key strategy for improving therapeutic outcomes (165, 166). By integrating advanced biotechnology, materials science, artificial intelligence, and precision medicine, we are poised to develop truly personalized hydrogel therapy systems.

Researchers have developed a personalized hydrogel system based on patient tumor genomic data (167–170). This system can customize the encapsulation of neoantigens and corresponding immunomodulators according to specific patients’ mutation and expression profiles. In a small-scale clinical trial, this strategy significantly improved treatment response rates, especially for patients unresponsive to conventional immunotherapy (171). Sun et al. reported an intelligent hydrogel system capable of dynamically responding to patients’ immune status. The specific chemical reactions between selected ligands and target analytes provide high selectivity in detecting complex fluids (172). Wang et al. developed a locally embedded photodynamic immunomodulatory DNA hydrogel for early warning and suppression of postoperative tumor recurrence. The DNA hydrogel containing PD-L1 aptamers, by integrating aptamer sensors, can capture and enrich *in situ* recurrent tumor cells and detect key cytokine levels in real-time, increasing local ATP concentrations to provide timely warning signals (173). Brandenberg et al. developed a high-throughput screening platform based on patient-derived organoids to evaluate the therapeutic efficacy of different hydrogel formulations. This system can simulate patient-specific tumor microenvironments *in vitro*, providing crucial basis for personalized hydrogel design (174). Studies have shown that hydrogel formulations screened through this method demonstrated better efficacy in subsequent clinical trials. Wei et al. proposed a dynamic hydrogel adjustment strategy based on circulating tumor DNA (ctDNA) monitoring (175). By periodically analyzing ctDNA in patients’ blood, this system can promptly detect resistance mutations and adjust drug combinations in the hydrogel accordingly. Preliminary clinical data suggest that this strategy significantly prolonged patients’ progression-free survival.

As research on personalized hydrogels advances, future efforts should focus on integrating genomics, transcriptomics, proteomics, and metabolomics data to develop a comprehensive platform for personalized hydrogel design. This holistic approach will further enhance the precision and efficacy of hydrogel-based therapies in breast cancer management, potentially revolutionizing treatment paradigms and improving patient outcomes.

## 7 Perspectives

In the future, researchers aim to develop hydrogel systems with active immunomodulatory functions capable of dynamically adjusting their properties based on individual patients’ physiological states to maximize biocompatibility and therapeutic efficacy. Moreover, by integrating technologies such as genomics, proteomics, and metabolomics, the development of hydrogel systems with active immunomodulatory capabilities will not only enhance therapeutic outcomes but also enable precise regulation of hydrogel degradation processes, thereby minimizing potential toxicity. This multifaceted approach represents a significant advancement toward personalized medicine, potentially revolutionizing breast cancer treatment strategies and improving patient outcomes.
